# Laparoscopic Versus Robotic Ventral Hernia Repair – An ACHQC Database 5-Year Analysis

**DOI:** 10.3389/jaws.2025.13352

**Published:** 2025-03-11

**Authors:** Diego L. Lima, Raquel Nogueira, Joao P. G. Kasakewich, Carlos Andre Balthazar da Silveira, Ana Caroline Dias Rasador, Sharon Phillips, Flavio Malcher

**Affiliations:** ^1^ Department of Surgery, Montefiore Medical Center, The Bronx, NY, United States; ^2^ Department of Surgery, Beth Israel Deaconess Medical Center, Harvard Medical School, Boston, MA, United States; ^3^ Bahiana School of Medicine and Public Health, Salvador, Bahia, Brazil; ^4^ Department of Biostatistics, Vanderbilt University Medical Center, Nashville, TN, United States; ^5^ Division of General Surgery, NYU Langone, New York, NY, United States

**Keywords:** robotic surgery, ventral hernia, minimally invasive surgery, laparoscopic surgery, incisional hernia

## Abstract

**Introduction:**

To compare laparoscopic and ventral hernia repair (VHR) in the last 5 years in the United States utilizing the Abdominal Core Health Quality Collaborative (ACHQC) database.

**Materials and Methods:**

A retrospective review of prospectively collected data from the ACHQC database was performed to include all adult patients that underwent laparoscopic and robotic VHR in the last 5 years. Univariate analysis was performed to compare outcomes from laparoscopic and robotic-assisted approaches across perioperative and postoperative outcomes.

**Results:**

ACHQC database identified 11,096 patients with midline hernias who underwent VHR with mesh. The Laparoscopic group with patients from 2018 to 2023 (LAP) had 2,063 patients, and the robotic group (ROBO) had 9,033 patients. There was no difference in sex, age, BMI, DM, smoking status and COPD between groups. Median hernia width was 4 cm (IQR 2–6) in the ROBO group and 3 cm (IQR 2–5) in the LAP group (p < 0.001). Incisional hernia was higher in the ROBO group 5,259 (58%) versus 1,099 (53%) in the LAP group (p < 0.001). Recurrent hernia was more common in the ROBO group when compared with the LAP group (p < 0.001). Both groups had more permanent synthetic mesh. Retromuscular repair was higher in the ROBO group, 3,201 (37.6%) versus 68 (4.2%) in the LAP group (p < 0.001). The intraperitoneal repair was higher in the LAP group 1,363 (83%) versus 2,925 (34%) in the ROBO group (p < 0.001) Transversus Abdominis Release (TAR) was higher in the ROBO group 1,314 (14.5%) versus 5 (0.2%) in the LAP group (p < 0.001). Fascial closure was higher in the ROBO group (8,649; 96.5% versus 1,359; 67.3% in the LAP group p < 0.001). Regarding mesh fixation, regular suture was higher in the ROBO group 92% versus 61% in the LAP group (p < 0.001). Tacks (p < 0.001) was higher in the LAP group. The ROBO group had more patients with an operative time of 240+ minutes when compared with the LAP group (p < 0.001). There was no difference in 30-days readmission rates, recurrence, reoperation, overall postoperative complications, 30-day SSI, SSO, seroma and SSOPI between the groups.

**Conclusion:**

The Robotic approach was associated with more technically challenging ventral hernia repairs with low complication rates over time. However, no differences in postoperative complications were found between the groups.

## Introduction

Ventral and incisional hernias are common with over 350,000 procedures performed annually in the United States [1–3]. Robotic-assisted minimally invasive surgery (MIS) for abdominal wall reconstruction has been facing a fast adoption worldwide growing at an exponential rate [4–6]. The laparoscopic repair faces challenges such as inability to properly close the fascial defect especially in large ventral hernias, limited dexterity to perform complex component separation techniques and limited options for mesh fixation [7].

The robotic platform offers clear advantages over laparoscopy. The three-dimensional view of the surgical field and articulating instruments with increased degrees of freedom allow surgeons to perform more precise movements and, therefore, to perform more challenging minimally invasive repairs [8]. Furthermore, the increased access to the robotic platform increased the rate of repairs performed with MIS [9]. However, the clinical efficacy and advantages of robotic hernia repair over laparoscopic surgery is still debatable with similar postoperative outcomes [10].

Despite the natural interpretation that a more articulating system would provide more dexterity, it is still important to better understand how the robotic platform impacted ventral hernia repairs through the years. The aim of this study is to compare Robotic ventral hernia repair (VHR) to the laparoscopic approach through the analysis of the complexity of hernia repairs in the last 5 years in the United States utilizing the Abdominal Core Health Quality Collaborative (ACHQC) database.

## Methods

### Data Collection

The data for this study originated from the Abdominal Core Health Quality Collaborative (ACHQC) from January 2013 to March 2023. The ACHQC is a nationwide hernia registry; as of now, there are 450 participant surgeons across the United States from academic and private institutions. They prospectively enter the patient’s information, which is broadly categorized into demographics, preoperative information, operative details, and postoperative details with patient-reported outcomes (PROs). As of early 2024, there are a total of 126,500 patients listed in the database who underwent ventral, lateral, and inguinal hernia repairs.

### Population and Comparison Groups

We selected elective laparoscopic ventral hernia repair with mesh and robotic-assisted ventral hernia repairs from January 2018 to December 2023. We excluded patients with concomitant hernias, repairs in contaminated and dirty fields, repairs with no mesh or patients with prior mesh. We also excluded patients who had no 30-day follow-up data.

### Outcomes

The primary outcome of our study is to assess Length of stay, SSO, SSI, seroma formation, readmission at 30 days after surgery using the ACHQC database.

### Statistical Analysis

Categorical variables were presented as frequency and percentage and compared among groups using Person Chi-squared test or Fisher exact test, and continuous variables were presented as median and interquartile range (IQR) and compared among groups using Kruskal-Wallis test or Wilcoxon rank sum tests.

### Institutional Review Board

This study was waived by our institutional review board (IRB).

## Results

ACHQC database identified 11,096 patients with midline hernias who underwent VHR with mesh from 2018 to 2023. The Laparoscopic (LAP) group with patients had 2,063 patients, and the robotic (ROBO) group had 9,033 patients. There was no difference in sex, age, BMI, DM, smoking status and COPD between groups. The baseline characteristics are listed in Table 1.

**TABLE 1:** Sociodemographic characteristics.

	Laparoscopic 2018–2023	Robotic 2018–2023
	N = 2063	N = 9,033
Sex		
Male (%)	1,102 (53%)	4,750 (53%)
Female (%)	961 (47%)	4,283 (47%)
Race		
White (%)	1,656 (81%)	7,057 (80%)
Non-White (%)	389 (19%)	1,805 (20%)
Age (IQR)	58 (47–68)	58 (47–68)
Current Smoker (%)	220 (10.7%)	874 (9.7%)
BMI (IQR)	32 (27–36)	32 (28–36)
Diabetes (%)	323 (16%)	1,489 (16%)
HTN (%)	883 (43%)	4,468 (49%)
Hepatic insufficiency or Liver Failure (%)	32 (1.55%)	63 (0.7%)
Ascites (%)	10 (0.5%)	24 (0.3%)
Dialysis (%)	10 (0.5%)	39 (0.4%)
COPD (%)	96 (4.7%)	417 (4.6%)
Imunnosupression (%)	112 (5.4%)	273 (3%)

IQR, Interquartile range; BMI, Body mass index; COPD, Chronic obstructive pulmonary disease.

Median hernia width was 4 cm (IQR 2–6) in the ROBO group and 3 cm (IQR 2–5) in the LAP group (p < 0.001). Median hernia length higher in the ROBO group 5 cm (IQR 2.5–10) versus 3 cm (IQR 2–5) in the LAP group. Incisional hernia was higher in the ROBO group 5,259 (58%) versus 1,099 (53%) in the LAP group (p < 0.001). Recurrent hernia was more common in the ROBO group when compared with the LAP group (p < 0.001) (Table 2) Both groups had more permanent synthetic mesh. Retromuscular repair was higher in the ROBO group, 3,201 (37.6%) versus 68 (4.2%) in the LAP group (p < 0.001). The intraperitoneal repair was higher in the LAP group 1,363 (83%) versus 2,925 (34%) in the ROBO group (p < 0.001). Transversus Abdominis Release (TAR) was higher in the ROBO group 1,314 (14.5%) versus 5 (0.2%) in the LAP group (p < 0.001). Fascial closure was higher in the ROBO group (8,649; 96.5% versus 1,359; 67.3% in the LAP group p < 0.001). Regarding mesh fixation, regular suture was higher in the ROBO group 92% versus 61% in the LAP group (p < 0.001). Tacks (p < 0.001) was higher in the LAP group (Table 2) The RobO group had more patients with an operative time of 240+ minutes when compared with the LAP group (p < 0.001). There was no difference in intraoperative complications between the groups (Table 2). Median LOS was similar in both groups with patients going home in the immediate postoperative time, however, laparoscopic approach was associated with a higher IQR and patients going home postop day 1 or 2 (p < 0.002).

**TABLE 2:** Intraoperative outcomes.

	Laparoscopic 2018–2023	Robotic 2018–2023	P-value
	N = 2,063	N = 9,033	
Operative time			<0.001
0–59 min (%)	762 (36.9%)	1,486 (16.5%)	
60–119 min (%)	955 (46.3%)	3,201 (35.5%)	
120–179 min (%)	247 (12%)	2,216 (24.5%)	
180–239 min (%)	68 (3.3%)	1,102 (12.2%)	
>240 min (%)	31 (1.5%)	1,025 (11.3%)	
Hernia Type			
Incisional (%)	1,099 (53%)	5,259 (58%)	<0.001
Parastomal (%)	68 (3.3%)	276 (3.1%)	0.57
Epigastric (%)	195 (9.5%)	920 (10.2%)	0.32
Umbilical (%)	721 (35%)	3,159 (35%)	0.99
Lumbar (%)	8 (0.39%)	68 (0.75%)	0.07
Spigelian (%)	85 (4.1%)	235 (2.6%)	<0.001
Diastasis (%)	50 (2.4%)	703 (7.8%)	<0.001
EHS classification			
M1	0%	0%	1
M2	195 (9.5%)	920 (10.2%)	0.32
M3	721 (35%)	3,159 (35%)	0.99
M4	0%	0%	1
M5	0%	0%	1
L1	0%	0%	1
L2	85 (4.2%)	235 (2.6%)	<0.001
L3	0%	0%	1
L4	8 (0.39%)	68 (0.75%)	0.07
Hernia characteristics			
Hernia Length – cm (IQR)	3 (2–5)	5 (2.5–10)	<0.001
Hernia Width – cm (IQR)	3 (2–5)	4 (2–6)	<0.001
Recurrent (%)	331 (16%)	1,825 (20%)	<0.001
Active Infection (%)	4 (0.19%)	22 (0.24%)	0.67
Non-elective (%)	41 (2%)	82 (0.9%)	<0.001
Mesh use (%)	1,868 (90.5%)	8,796 (97.4%)	<0.001
Biological tissue-derived (%)	52 (2.5%)	51 (0.6%)	
Permanent synthetic (%)	1804 (87.4%)	8,587 (95.1%)	
Resorbable synthetic (%)	11 (0.5%)	150 (1.7%)	
Mesh location			<0.001
Inlay (%)	40 (1.9%)	120 (1.3%)	
Onlay (%)	189 (9.2%)	153 (1.7%)	
Sublay (%)	1,639 (79.4%)	8,520 (94.3%)	
Retromuscular (%)	68 (3.3%)	3,201 (35.4%)	<0.001
Preperitoneal (%)	221 (10.7%)	2,748 (30.4%)	<0.001
Intraperitoneal (%)	1,363 (66.1%)	2,925 (32.4%)	<0.001
Mesh fixation			
Sutures (%)	1,097 (53.2%)	5,590 (61.9%)	<0.001
Tacks (%)	1,608 (77.9%)	264 (2.9%)	<0.001
Adhesives (%)	21 (1%)	470 (5.2%)	<0.001
Staples (%)	9 (0.4%)	4 (0.04%)	<0.001
Myofascial release (%)	41 (2%)	2,986 (33%)	<0.001
External oblique (%)	1 (0.05%)	1 (0.01%)	<0.001
Transversus abdominis (%)	5 (0.2%)	1,314 (14.5%)	<0.001
Retrorectus sheath (%)	35 (1.7%)	2,704 (29.9%)	0.5
Fascial closure (%)	1,359 (67.3%)	8,649 (96.5%)	<0.001
Intraoperative complications	13 (0.63%)	85 (0.94%)	0.17
Intraoperative hemorrhage	0 (0%)	2 (0.02%)	0.58
Bowel injury (%)	11 (0.5%)	53 (0.6%)	0.12
Bladder injury (%)	0 (0%)	6 (0.07%)	0.32
Gastric injury (%)	1 (0.05%)	0 (0%)	0.01
Vascular injury (%)	0 (0%)	1 (0.01%)	0.69
Peritoneal access injury (%)	0 (0%)	6 (0.07%)	0.32

Cm, Centimeters; IQR, Interquartile range.

There was no difference in 30-days readmission rates, recurrence, reoperation, overall postoperative complications, 30-day SSI, SSO, seroma and SSOPI between the groups (Table 3).

**TABLE 3:** Perioperative outcomes.

	Laparoscopic 2018–2023	Robotic 2018–2023	P-value
	N = 2063	N = 9,033	
Readmission (%)	50 (2.4%)	231 (2.6%)	0.73
Recurrence (%)	9 (0.4%)	20 (0.2%)	0.085
Reoperation (%)	12 (0.6%)	82 (0.9%)	0.14
Reoperation for recurrence (%)	3 (0.15%)	9 (0.01%)	0.19
Overall postoperative complications (%)	207 (10%)	913 (10%)	0.92
SSI (%)	16 (0.8%)	55 (0.6%)	0.39
Superficial (%)	9 (0.4%)	32 (0.35%)	0.89
Deep (%)	3 (0.15%)	21 (0.2%)	0.15
Organ space (%)	5 (0.2%)	6 (0.07%)	0.048
SSO (%)	132 (6.4%)	622 (6.9%)	0.43
SSOPI (%)	143 (6.9%)	669 (7.4%)	0.46
Cellulitis (%)	9 (4.3%)	17 (1.9%)	0.03
Non-healing wound (%)	0 (0%)	4 (0.4%)	0.34
Fascial disruption (%)	0 (0%)	3 (0.3%)	0.41
Seroma (%)	107 (5.2%)	510 (5.6%)	0.28
Hematoma (%)	9 (0.4%)	63 (0.7%)	0.18
Enterocutaneous fistula (%)	1 (0.05%)	2 (0.02%)	0.51

SSI, Surgical site infection; SSO, Surgical site occurrence; SSOPI, Surgical site occurrence requiring intervention.

## Discussion

Our study demonstrated that the robotic-assisted surgery had higher rates of fascial closure, myofascial release, and mesh fixation using suture when compared to the laparoscopic group. Furthermore, the robotic group had higher median hernia width and length than the laparoscopic group. There was no difference in 30-day SSI, SSO, seroma formation and SSOPI between the groups. We aimed to compare the last 5 years of laparoscopic ventral hernia repair with the robotic approach. Laparoscopic surgery is well established with surgeons already with extensive experience while the robotic assisted technique still is new for a considerable number of surgeons in the United States with access to the robotic platforms more recently.

A recent meta-analysis comparing robotic and laparoscopic ventral hernia repair with 5 studies and 3,732 patients showed that the robotic approach was associated with longer operative time and no differences in SSO and recurrence [10]. This is in accordance with our study. We found that the robotic approach had more cases with more than 240 min. Since most laparoscopic cases were intraperitoneal mesh repair with the use of tacks and lower rates of fascial closure, it makes sense that these cases were less challenging to surgeons. Other studies have also demonstrated increased OT with the robotic platform [11, 12].

Howard et al. analyzing trends in ventral hernia repair in the United States from 2007 to 2015 observed an increase in MIS techniques, component separation and mesh use [1]. Other studies also observed the increased adoption of these techniques [13, 14]. Madion et al. found a 2-fold increase in MIS techniques for ventral hernia repair with stable wound morbidity in all MIS modalities and decreased wound morbidity when compared to open surgery [13]. A review of the Michigan Surgical Quality Collaborative showed an increased proportion of robotic ventral hernia repair up to 22.5% between 2012 and 2018 [4].

The Robotic approach has become popular among surgeons due to possibility to perform more complex repairs in a minimally invasive fashion with decreased postoperative wound morbidity. Some techniques that were previously only able to be mainly performed by open technique such as retromuscular mesh repair with transversus abdominis release can now be performed with the robotic platform and with similar wound morbidity and lower length of stay [15–18]. Henriksen et al (2024) performed a retrospective propensity score matching study comparing robotic RM (retromuscular) repair with laparoscopic IPOM (intraperitoneal onlay mesh) repair with 1,136 patients from the Danish Hernia Database. The authors found that for primary ventral hernias IPOM repair had a higher group of smaller mean vertical defect size when compared to the robotic RM repair. Furthermore, authors found that the laparoscopic IPOM was associated with longer mean LOS and higher rates of readmissions within 90 days [19]. This is in accordance to our study where median LOS was the same for both groups with laparoscopic group having more outliers in the postoperative day 1 or 2 when compared to the robotic approach.

The significance of haptic feedback in robotic surgery remains a topic of debate. Early 2000s research on robotic systems suggested that the absence of haptic feedback might contribute to unintentional tissue injury [19, 20]. However, skilled surgeons can compensate by relying on visual cues, such as tissue deformation, to assess force [21, 22]. Meccariello et al. demonstrated that experienced surgeons more accurately identified the thickness of custom-made membranes without haptic feedback compared to junior surgeons [23]. To date, no studies have examined the impact of missing haptic feedback on tissue trauma in abdominal wall surgery, and our findings do not indicate any difference in outcomes [24].

### Limitations of the Study

This study has several limitations. It is a retrospective study with prospective data entered by the surgeons who input their data into the ACHQC database. This may lead to recall bias. Second, a performance bias might be present as surgeons interested in hernia surgery are more likely to participate in data collection, which was highlighted by the increased proportion of robotic procedures compared to conventional laparoscopy. Additionally, we lack long-term follow-up data which limits our ability to comment on important factors such as long-term recurrence. Furthermore, our study did not perform an analysis of individual surgeons’ performance, and we cannot directly evaluate increased dexterity with our data. Robotic-assisted surgery has improved 3D vision and tremor reduction when compared to the laparoscopic approach and a causality cannot be.

Lastly, the data is collected through voluntary self-reporting, so there may be selection bias if participating surgeons input only some of their cases. The strength of our study lies in our large sample size (n = 11,096).

## Conclusion

The Robotic approach was associated with more technically challenging ventral hernia repairs with low complication rates over time. However, no differences in postoperative complications were found between the groups.

## Data Availability

The raw data supporting the conclusions of this article will be made available by the authors, without undue reservation.
